# Impacts of radiation exposure, hindlimb unloading, and recovery on murine skeletal muscle cell telomere length

**DOI:** 10.1038/s41526-023-00303-1

**Published:** 2023-09-15

**Authors:** Elisia D. Tichy, Ji-Hyung Lee, Grant Li, Katrina N. Estep, F. Brad Johnson, Foteini Mourkioti

**Affiliations:** 1 Department of Orthopaedic Surgery, Perelman School of Medicine, University of Pennsylvania, Philadelphia, PA 19104 USA; 2 Department of Pathology and Laboratory Medicine, Institute on Aging, Perelman School of Medicine, University of Pennsylvania, Philadelphia, PA 19104 USA; 3 Department of Cell and Developmental Biology, Perelman School of Medicine, University of Pennsylvania, Philadelphia, PA 19104 USA; 4 Penn Institute for Regenerative Medicine, Musculoskeletal Program, Perelman School of Medicine, University of Pennsylvania, Philadelphia, PA 19104 USA

**Keywords:** Stem cells, Cell biology

## Abstract

Astronauts are exposed to harsh conditions, including cosmic radiation and microgravity. Spaceflight elongates human telomeres in peripheral blood, which shorten upon return to Earth and approach baseline levels during postflight recovery. Astronauts also encounter muscle atrophy, losing up to 20% loss of muscle mass on spaceflights. Telomere length changes in muscle cells of astronauts remain unexplored. This study investigates telomere alterations in grounded mice experiencing radiation exposure and muscle atrophy, via a hindlimb unloading spaceflight mimicking model. We find telomere lengthening is present in muscle stem cells and in myofiber nuclei, but not in muscle-resident endothelial cells. We further assessed telomere length in the model following hindlimb unloading recovery. We find that telomere length failed to return to baseline values. Our results suggest a role for telomeres in muscle acclimatization, which is relevant for the well-being of astronauts in space, and upon their return to Earth.

## Introduction

In the vacuum of space, astronauts are continuously bombarded by high energy cosmic radiation and must perform missions in microgravity^1^. A better understanding of the consequences of these two factors on body function and physiology are crucial for the planning of safe and successful long-duration future missions. A major detriment experienced by astronauts in flight is muscle atrophy, where a 1 month spaceflight can reduce muscle mass by 20% and muscle strength by 30%^2^. Even with currently implemented spaceflight countermeasures, involving rigorous exercise, the deleterious physiological effects of spaceflight in muscle atrophy and weakness induction are not completely abrogated^3–5^. In addition to microgravity, astronaut exposure to cosmic radiation can have effects on body systems that are still being elucidated. It is well established that the skeletal muscle atrophy due to exposure to both microgravity and radiation is problematic because of its rapid onset and severity^6^.

Muscle stem cells (MuSCs), also called satellite cells, are necessary for muscle formation in embryogenesis and for muscle growth during childhood and adolescence^7,8^. In healthy adults, MuSCs from undamaged muscles remain quiescent. Following injury, such as that arising from strenuous exercise, MuSCs become activated, proliferate, and differentiate in a series of coordinated steps, to properly repair muscle damage^9^. In the context of muscle atrophy, MuSC properties are less clear. Atrophy caused by cancer cachexia is known to activate specific signaling pathways in skeletal muscles related to inflammation, protein synthesis, and autophagy^10^. In satellite cells, atrophy-induced signaling exerts effects on MuSC proliferative potential and differentiation capacities^11,12^. In terms of effects of radiation exposure to MuSCs, limited data are available. Exposure of cultured human muscle progenitor cells to ionizing radiation has negative effects on proliferative capacity and alters inflammatory signaling^13^. Studies using chronic, lower dose, whole-body irradiation of mice resulted in reduced MuSC numbers, reduced proliferative capacity, and increased quiescence, but it had little effect on differentiative capacity^14^. Higher dose leg-specific radiation exposures have demonstrated similar results on reduced proliferative capacities of juvenile mice; however, myofiber sizes and MuSC differentiation effects were present as well^15^, suggesting that level and duration of exposure could have different effects on MuSCs, depending on these factors.

The length of telomeres is an important parameter of tissue proliferative potential^16^. Telomeres are repetitive sequences located at the ends of chromosomes, which can function as cellular proliferation clocks^17^. Telomere attrition leads to loss of cellular functionality and can lead to stem cell exhaustion in diseases such as Duchenne muscular dystrophy^18,19^. Shortened telomeres are also correlated with atrophy of many organ compartments, including brain^20^, spleen, small intestine, and reproductive system, among others^21^. Additionally, it has been reported that telomeres in cultured mouse and human fibroblasts appear more susceptible than other regions of the genome to DNA damage, and they exhibit a persistent DNA damage response following X-ray irradiation^22^. Increased DNA damage found at telomeres has been associated with telomere attrition and/or uncapping and cellular senescence, leading to loss of proper cellular functionality^23,24^.

Recently, the effects of spaceflight have been meticulously investigated in the NASA Twins Study, where effects of preflight, inflight, and postflight conditions on a monozygotic twin were measured by multiple metrics and compared with the grounded twin brother^25^. That study revealed telomere lengthening in T cells of the inflight astronaut, which rapidly shortened postflight^25^. Another study investigating different astronaut flight conditions found similar results^26^. However, despite the importance of maintaining skeletal muscles during spaceflight, telomere length metrics in astronaut muscles have not been assessed.

The goal of this study was to measure telomere length in resident skeletal muscle cells of grounded, wild-type mice experiencing spaceflight-like conditions, using a whole-body irradiation (IR) and hindlimb suspension (HLS)/mechanical unloading approach. We found that a combination of IR and HLS resulted in telomere elongation, specifically in myogenic cell populations, which did not recover upon a short duration reloading of hindlimbs.

## Results

### Effects of HLS and IR exposure on skeletal muscle properties

**Fig. 1:**
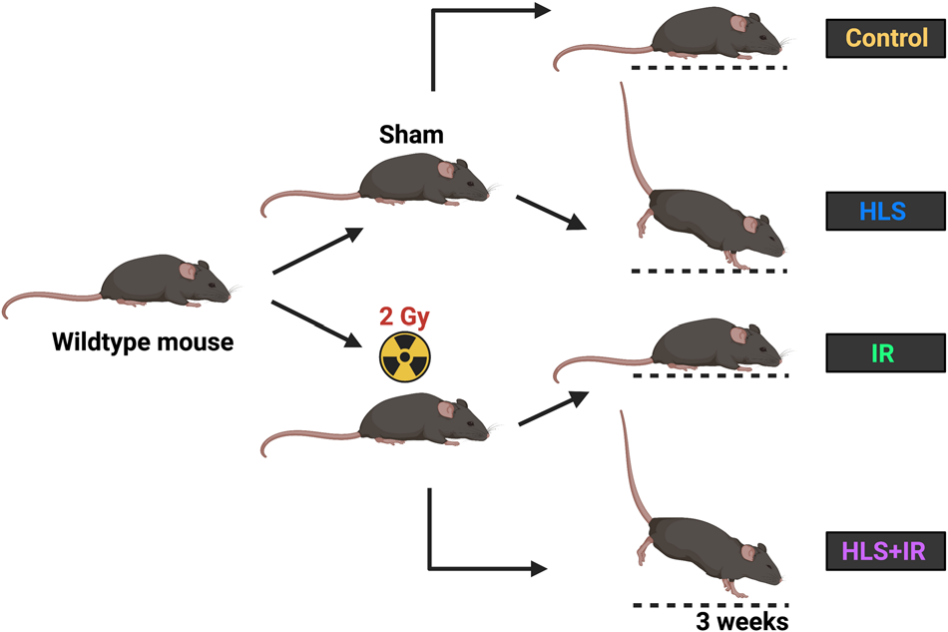
Schematic of experimental groups used for the study. Mice were pre-exposed to 2 Gy whole-body irradiation or sham irradiated and further divided into groups experiencing HLS or not. HLS hindlimb suspension, IR ionizing radiation.

**Fig. 2:**
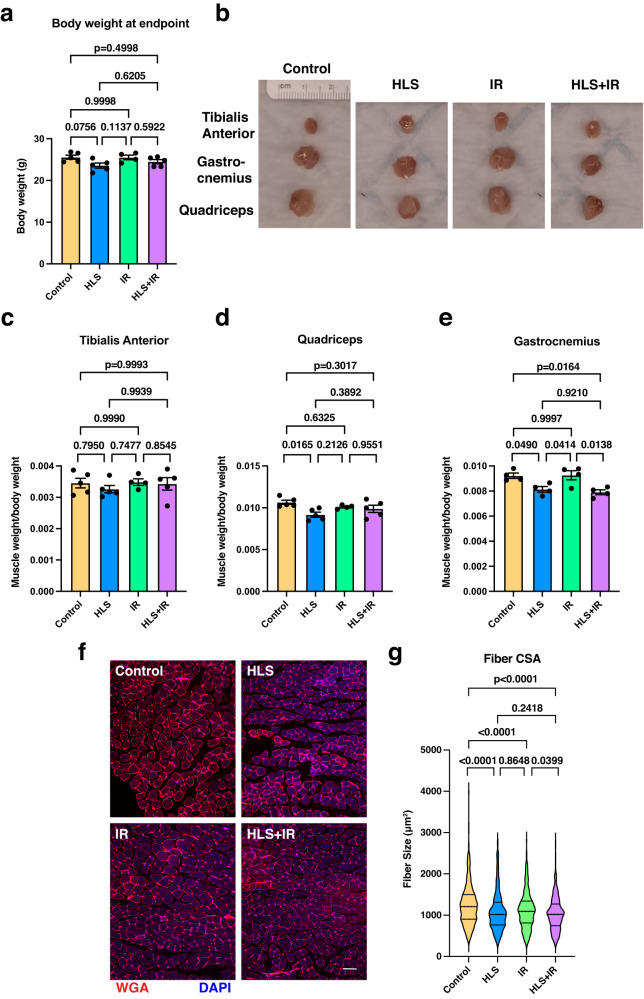
Hindlimb suspension affects gastrocnemius muscles. **a** Assessment of mouse body weights at the end of the experimental study show no gross difference between groups. **b** Images of dissected hindlimb muscles (tibialis anterior, gastrocnemius and quadriceps) from all groups. **c**–**e** Ratio of gross muscle weights, divided by the total body weight of the respective mouse. **c** tibialis anterior, **d** quadriceps, **e** gastrocnemius. **f** Representative images of gastrocnemius muscle fibers stained with fluorescently labeled WGA, which marks muscle fiber boundaries. Scale bar: 100 μm. **g** Enumeration of muscle fiber cross-sectional areas (CSA) from gastrocnemius muscles. At least 4 mice per condition were analyzed. Represented are mean ± SEM for column graphs and median with 25th and 75th quartiles marked for violin plots. Data were analyzed using one-way ANOVA and Tukey’s multiple comparison test of means. Adjusted *p* values are displayed.

To address the effects of muscle atrophy and/or irradiation (IR) on skeletal muscles, we utilized the widely accepted hindlimb suspension (HLS) ground-based model^27^, in combination with or in the absence of whole-body ionizing radiation exposure. In our experimental design, mice were divided into four different cohorts: control (without IR exposure, without hindlimb suspension (HLS)), HLS only (no IR), IR only (no HLS), and combined (HLS + IR); (Fig. 1). More specifically, wildtype mice were pre-exposed to 2 Gy whole-body irradiation or sham irradiated and further divided into groups experiencing HLS or not. Following a 3-week time course, mice were sacrificed, and various tissue weight metrics were obtained. We found that at the study endpoint, mouse whole-body weights were similar for all groups (Fig. 2a), demonstrating that HLS did not detrimentally affect mouse appetite. This finding is especially important, since hindlimb unloading has been used as method to study behavioral despair and depression, and appetite changes, if any, could have influenced our study results^28^.

We next determined the effects of our experimental design on internal organs. We found IR exposure alone had no effects on kidney or spleen weights, but it did cause significant (*p* < 0.0001) reductions in testes weights, when compared to sham/control mice (Supplementary Fig. 1a–d). These findings are in agreement with previous studies in mice and humans exposed to IR^29,30^. HLS also had deleterious effects on testes gross size and normalized weight (Supplementary Fig. 1a, d; *p* < 0.0001), consistent with a previous report^31^. Additionally, kidney weights in HLS and HLS + IR groups were slightly increased compared with controls (Supplementary Fig. 1a, c; *p* = 0.0452, 0.0705, respectively), a finding that was similarly reported in a rat HLS model^32^. No differences were found in spleen and kidney weights in combinatorial HLS + IR versus respective controls; however, the testes HLS + IR group displayed additive detrimental effects on testicular weight, relative to individual groups, consistent with prior literature on organ size changes from HLS and/or IR exposures^29–31^.

To assess the effects of these experimental conditions on skeletal muscles, muscles were dissected post-trial and the level of atrophy was queried (Fig. 2b). We found little difference in tibialis anterior weights from mice that underwent HLS, and quadriceps weights were variable, compared to respective controls (Figs. 2b–d). However, gastrocnemius muscles did exhibit a significant muscle atrophy in HLS and HLS + IR groups (Fig. 2b, 2e; *p* = 0.0490, *p* = 0.0138, respectively), leading us to focus on this muscle for the duration of the study. To confirm that gastrocnemius weight reductions affected muscle fibers, muscles were cryosectioned and stained with fluorescently-conjugated wheat germ agglutinin (WGA), which labels extracellular matrix regions^33^. We observed significantly smaller fiber sizes in both HLS and HLS + IR groups, relative to control and IR groups (Fig. 2f, g; *p* < 0.0001, *p* = 0.0399, respectively), further validating the muscle atrophy effect. In aggregate, these data unequivocally demonstrate a positive correlation between HLS and gastrocnemius muscle atrophy that agrees with the muscle wasting seen in previous HLS models^27^ and which mimics that described in astronauts during spaceflights^34^.

### Telomere length dynamics in spaceflight mimicked muscles

**Fig. 3:**
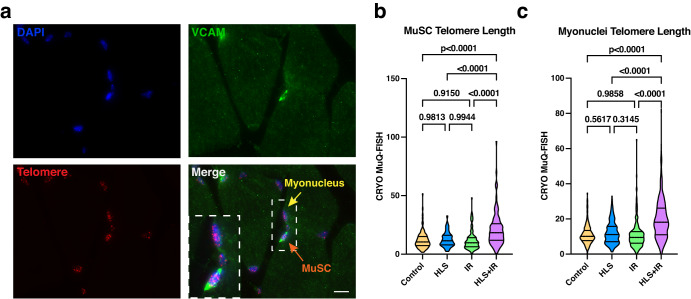
Telomere length assessments in gastrocnemius myogenic cells. **a** Representative images of MuSCs (VCAM + , in the satellite cell position; green), telomere staining (PNA probe; red) and nuclear staining (DAPI; blue). Myonuclei are nuclei residing within the myofibers and do not stain for VCAM. Scale bar: 10 μm. **b** Quantification of telomere length in MuSC populations of gastrocnemius muscles from control, HLS, IR, and HLS + IR mice. Telomere length was calculated as the total telomere signal intensities divided by the intensity of DAPI, to account for cell cycle positioning. **c** Myonuclei telomere length evaluation in gastrocnemius muscles. Three mice per experimental group were analyzed. Violin plots in this figure are depicting median with 25th and 75th quartiles. Data were analyzed using one-way ANOVA and Tukey’s multiple comparison test of means. Adjusted *p* values are displayed.

In the Twins Study, Garrett-Bakelman et al. demonstrated a significant lengthening of telomeres in circulating blood cells (T cells) in the astronaut twin while in space^25^. However, telomere lengths in muscle cells were not measured. Using a HLS + IR spaceflight-mimicking system, we wished to query the effects of telomere length on several skeletal muscle-resident cell types. First, we performed CRYO MuQ-FISH staining of telomeres on gastrocnemius cryosections^35^. With this method, relative telomere length was determined by intensity measurements of a fluorescently labeled telomeric peptide nucleic acid probe, normalized to the fluorescence intensity staining of the entire nucleus by DAPI staining. Cells of interest were identified by additional immunostaining to mark desired cells and/or the cellular positions within the muscle. We first queried telomere length in MuSCs, defined as VCAM+ cells that appear in the satellite cell position (Fig. 3a). Quantification of telomere length revealed no changes between control, HLS, or IR MuSCs (Fig. 3b). However, when HLS + IR MuSC telomeres were assessed, we found significant telomere lengthening, as compared to all other groups (Fig. 3b, *p* < 0.0001). To evaluate the cellular consequences of these alterations, we evaluated MuSC populations and properties. We first assessed whether there were changes in MuSC numbers in atrophied muscles, as had been suggested previously^12^. Gastrocnemius cryosections were stained with the MuSC marker, Pax7^8^, and total number of positively stained cells per unit area was queried. We found no changes in MuSC numbers between control vs IR, and control vs HLS + IR treated groups, while a significant increase in MuSC numbers was observed in the control vs HLS only group (Supplementary Fig. 2a, b; *p* < 0.0001). While these findings are interesting and unexpected, they did not correlate with the observed changes in telomere length of the HLS + IR group. To exclude other cellular defects associated with telomere length^17,36^, we also investigated whether MuSCs were undergoing senescence in response to our experimental manipulations. To interrogate the level of senescence in skeletal muscles in our study, gastrocnemius cryosections were stained for senescence-associated β-galactosidase activity. We found no correlation between positively stained regions and cells within the satellite cell position (Supplementary Fig. 3). These findings make senescence unlikely as an influencing factor of telomere length modulation in HLS + IR MuSCs.

It is known that skeletal muscle mass change during unloading is associated with altered myonuclei^37^. Therefore, we next evaluated whether telomere lengthening in HLS + IR gastrocnemius muscles was specific to only MuSCs or whether additional myogenic nuclei also exhibited this phenomenon. To this end, myonuclei, which were defined as localizing within muscle fibers and staining negative for the stem cell marker VCAM^38^, were examined for telomere length changes. Similar to what was found for MuSCs, myonuclei also displayed increased telomere length in HLS + IR gastrocnemius muscles, when compared with control, HLS, or IR gastrocnemius myofiber myonuclei (Fig. 3c; *p* < 0.0001). These data demonstrate that combinational HLS + IR of rodents on the ground affects gastrocnemius myogenic cell telomere length.

**Fig. 4:**
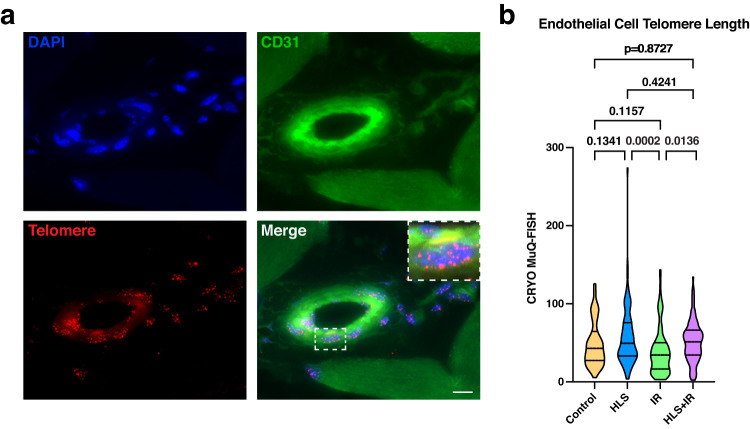
Telomere length quantification of non-myogenic muscle-resident cells. **a** Representative image of endothelial cell (CD31 + ; green), telomere staining (PNA probe; red), and nuclei (DAPI; blue). Scale bar: 10 μm. **b** Calculated telomere length of endothelial cells in gastrocnemius muscles from control, HLS, IR, and HLS + IR mice. Three mice per experimental group were analyzed. Violin plots are depicting median with 25th and 75th quartiles. Data were analyzed using one-way ANOVA and Tukey’s multiple comparison test of means. Adjusted *p* values are displayed.

Since mechanical forces, including changes in gravity, have been shown to affect endothelial cells^39^, we subsequently assessed whether telomere length increases in HLS + IR mice were also observed in endothelial cells residing in the gastrocnemius muscle of hindlimb unloaded mice with and without radiation exposure. CRYO MuQ-FISH was performed using the CD31+ (also known as PECAM-1) marker, previously shown to accurately label endothelial cells^40^. We observed telomere length dynamics to be quite variable between experimental groups in endothelial cells (Fig. 4a, b), demonstrating that effects of HLS + IR were most pronounced and reproducible in cells deriving from a myogenic lineage.

Altogether, these results demonstrated that telomere length is increasing synergistically in response to low gravity and irradiation only in muscle cells (MuSCs and myonuclei) but not in endothelial cells within skeletal muscles.

### Effects of muscle reloading on muscle telomere length

**Fig. 5:**
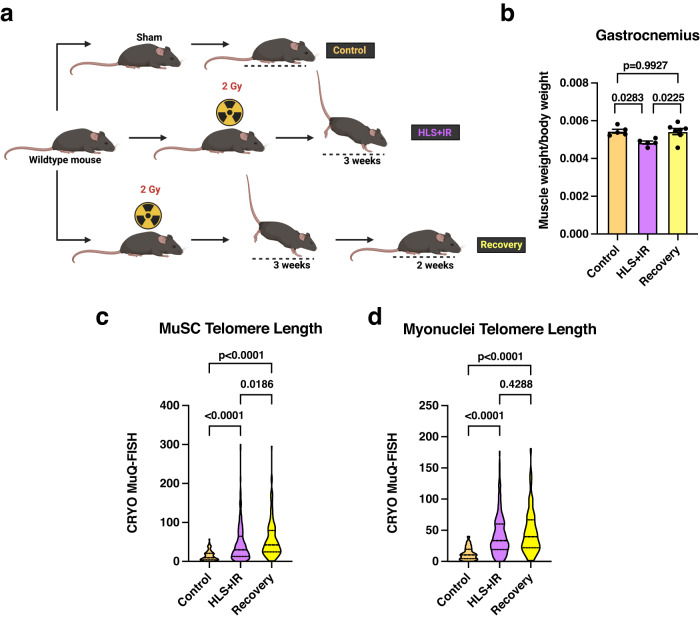
Gastrocnemius properties following hindlimb reloading. **a** Schematic of experimental groups. Recovery mice were reloaded for 2 weeks prior to harvesting. **b** Gastrocnemius muscle weights, normalized to respective mouse total body weight. **c** Telomere length assessments of MuSCs in control, HLS + IR, or recovery gastrocnemius cryosections. **d** Myonuclei telomere length assessments in control, HLS + IR and recovery gastrocnemius muscles. At least 3 mice per condition were analyzed. Represented are mean ± SEM for column graphs and median with 25th and 75th quartiles marked for violin plots. Data were analyzed using one-way ANOVA and Tukey’s multiple comparison test of means. Adjusted *p* values are displayed.

In the NASA Twins Study, it was shown that while spaceflight caused telomere lengthening in some peripheral blood cell populations, return to earth caused rapid telomere shortening within 48 hours, and a return to near baseline levels within months^25^. Since our data demonstrated telomere lengthening in HLS + IR MuSCs, we next sought to determine whether telomere length in these cells also shortened following a recovery period, where mice were returned to conventional mobility. To assess this possibility, HLS + IR mice were allowed to recover for 2 weeks, with unrestricted movement and normal muscle loading. For this arm of the study, control, HLS + IR, and recovery groups were compared (Fig. 5a). First, we assessed normalized organ weights and found no significant changes to normalized spleen weights (Supplementary Fig. 4a) and kidney weights (Supplementary Fig. 4b) between the groups. Interestingly, testes weights also trended higher in recovered animals, but they remained significantly smaller when compared to control weights (Supplementary Fig. 4c; *p* < 0.0001). Together, these data confirm that the recovery did not interfere with general organ mass. In assessing the effects of recovery on skeletal muscles, we observed that gastrocnemius muscles of recovered mice were larger than HLS + IR mice and were of similar size to controls (Fig. 5b; *p* = 0.9927), demonstrating that 2 weeks post-unloading is sufficient to regain normal skeletal muscle size. To evaluate telomere length alterations in the recovered muscles, we performed CRYO MuQ-FISH in the gastrocnemius-derived MuSC populations and myonuclei between the experimental groups. Surprisingly, we did not observe telomere shortening in the recovery MuSCs, but instead they retained elongated telomere length and continued to increase even further, relative to the HLS + IR group (Fig. 5c; *p* = 0.0186). Similarly, telomere length in myonuclei also remained elongated following recovery (Fig. 5d), which also trended higher but was not significantly different compared with the HLS + IR group (*p* = 0.4288). To further delineate whether there were differential telomere length changes in muscle cell populations, which may have been buried by the averaging of telomere length analysis, we plotted this dataset as a distribution on a per cell basis (Supplementary Fig. 5). These data show, that unlike what was reported for T cells in the Twins Study, where a proportion of the return to Earth cells exhibited population of critically shortened telomeres^25^, we found few MuSCs with very short telomeres and many more MuSCs with long telomeres. The telomere length distribution of muscle cells is somewhat different from the distributions reported in a follow-up study of blood cells in populations of short and long telomeres^26,41^, suggesting that different cell types might have different mechanisms of managing telomere length under spaceflight or spaceflight-mimicking conditions. In aggregate, these data demonstrate that hindlimb reloading for the indicated recovery period was not sufficient to shorten telomere length to control levels.

### Telomere lengthening mechanisms in radiation-exposed unloaded muscles

**Fig. 6:**
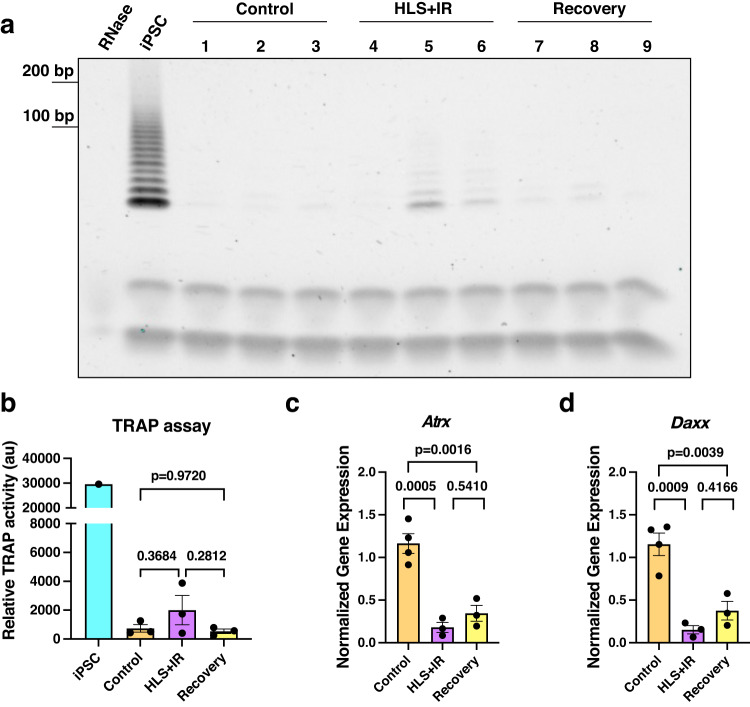
Interrogation of telomere lengthening mechanisms in hindlimb unloading and recovery. **a** TRAP assay to assess telomerase activity in control, HLS + IR, and recovery skeletal muscle lysates. RNase treatment served as a negative control and induced pluripotent stem cell (iPSC) lysate served as a positive control. Each lane is from one gastrocnemius muscle lysate per mouse, with three biological replicates per group. **b** Quantification of telomere elongation via telomerase from **a**. Displayed are mean ± SEM. **c**, **d** Quantitative real-time PCR of ALT pathway regulators *Atrx* and *Daxx* from gastrocnemius muscles of control, HLS + IR, and recovery groups. *Gapdh* served as a housekeeping gene for normalization. At least three mice were examined per group. Displayed is mean ± SEM. Data were analyzed using one-way ANOVA and Tukey’s multiple comparison test of means. Adjusted *p* values are displayed.

The most common mechanism of telomere elongation is through the action of the enzyme telomerase^42^. To query the role of telomerase in the observed telomere lengthening of HLS + IR gastrocnemius muscle cells, we first assayed for its expression. *Tert,* the catalytic subunit of telomerase, was undetectable in skeletal muscles from control, HLS + IR, or recovery groups by qRT-PCR (Supplementary Fig. 6a). Similarly, expression of TERT was absent at the protein level, by immunohistochemistry of cryosections (Supplementary Fig. 6b). Because telomerase expression in muscles may be transient and/or only expressed in a small population of cells, we interrogated telomerase activity by a telomerase repeated amplification protocol (TRAP) assay. We found low telomerase activity in experimental gastrocnemius muscles (Fig. 6a), with no significant changes between control muscles and HLS + IR (*p* = 0.3684), control and recovery (*p* = 0.9720), or between HLS + IR and recovery groups (*p* = 0.2812; Fig. 6b). Our findings are in agreement with a prior study that showed telomerase activity is lost in differentiated skeletal muscle cells^43^, and further suggest that telomerase might not have a substantial role in telomere elongation in this system.

Since the observed telomere lengthening in muscles could not be explained by telomerase activity, we sought to identify other mechanisms by which telomere lengthening could be attributed. Alternative lengthening of telomeres (ALT) is a recombination-based telomere maintenance mechanism utilized by 10–15% of human cancers^44^. It has been previously reported that the DNA damage responding proteins, ATRX and DAXX, must be downregulated for ALT to occur^45–48^. To this end, we quantified the expression of these genes in control, HLS + IR, and recovery muscles. Interestingly, we observed significant downregulation of both *Atrx* and *Daxx* transcripts in HLS + IR muscles, relative control (*p* = 0.0005, *p* = 0.0009, respectively; Fig. 6c, d), suggesting that ALT-related pathways are engaged in this model. When recovery groups were examined, both *Atrx* and *Daxx* were still downregulated, although they did trend higher in both cases, relative to HLS + IR groups (*p* = 0.5410, *p* = 0.4166, respectively; Fig. 6c, d). Taken together, these data raise the possibility of ALT-related mechanisms being employed in the observed telomere lengthening in irradiated, unloaded muscles, as well as during reloading.

## Discussion

Astronauts experience adverse health effects on long-duration missions, including headaches, dizziness, congestion, changes in blood cells, and fatigue, as well as severe bone and muscle loss^49^. A better understanding of the causes of such impediments to explorer health in an earthbound model could serve beneficial effects in the development of additional countermeasures or prophylactic treatments meant to reduce physical health burdens. Recently, a comprehensive NASA study, also commonly referred to as the Twins Study, investigated and compared a multitude of metrics, including physical changes, of an earthbound monozygotic twin astronaut, to his brother, experiencing spaceflight^25^. One reported aspect of this study was an observed effect of telomere lengthening in peripheral blood T cells during spaceflight, which was absent in the grounded twin^25^. Outside of space, telomere length changes have been reported in degenerative diseases, which often coincide with tissue atrophy^50^, including skeletal muscle^18,19,51,52^. However, telomere length alterations in skeletal muscle cells, both from mice^18^ and humans^53,54^, are not correlated with the effects of aging, where sarcopenia, a muscle wasting process, is a hallmark^55^. Although muscle deterioration is a serious problem in astronauts’ health, the effect of gravity changes on telomeres of muscle cells has not been studied. In addition, the impact of the effects of radiation on telomere length are still inconclusive. A study of Chernobyl radiation cleanup workers demonstrated that occupational exposure to ionizing radiation led to telomere lengthening in these workers in peripheral blood leukocytes^56^. Similar effects were reported for atomic bomb survivors, but only those who were exposed to lower doses of radiation, as higher dose radiation exposure led to telomere attrition in peripheral blood T cells^57^. However, studies on telomere length following radiation exposure, specifically focusing on the muscle compartment, are nonexistent. Most importantly, the combined effect of weightlessness-induced skeletal muscle atrophy and cosmic radiation exposure reported during spaceflight^58^ led us to query whether these factors can contribute to changes in telomere dynamics in myogenic cells in a ground-based mouse model.

HLS in rodents is a common model used to study the effects of ground-based muscle atrophy, through mechanical unloading and skeletal muscle disuse^59^. Using this HLS strategy in mice, we observed ~20% reductions in gastrocnemius muscle, similar to what has been previously reported^59^. Despite this loss of muscle mass, no changes in telomere length in the HLS model alone were evident, suggesting that simulated weightless conditions alone are not sufficient to modify telomere length. To mimic cosmic radiation exposure in spaceflights, we exposed mice to whole-body ionizing radiation by itself or utilized a combined approach of HLS and irradiation. Under these conditions, we found telomere lengthening only in myogenic cell types in the HLS + IR group but IR exposure alone had minimal impact on telomeres. Intriguingly, endothelial cells present in the affected muscles do not exhibit significant telomere length changes in either group, suggesting that telomeres within skeletal muscle cells are more susceptible to telomere length changes in our experimental system. Overall, the telomeres in combined radiation exposure and simulated microgravity conditions demonstrated a synergistic telomere lengthening effect when applied together, specifically in myogenic cells but not in other cells with the muscle architecture.

Although many detrimental health effects are associated with telomere shortening^60,61^, a growing body of evidence suggests that very long telomeres may be problematic in human cells, as these telomeres could develop instabilities and telomeric fragility^62^, supporting the notion that tight control of telomere length homeostasis is essential. In line with this statement, in the Twins Study, the return to ground astronaut demonstrated a rapid shortening of telomere length, within 48 h after landing, to levels below earthbound twin, and telomere length extended but still remained shorter than the earthbound twin, even after 9 months^26^. Similar findings were found in other astronaut studies as well^26,41^. To query whether telomere shortening occurred within skeletal muscle cells of our experimental design, we implemented a 2-week recovery period, where mechanical loading of hindlimb muscles was reestablished. The longer 2-week endpoint compared to the initial 48 h used in the Twins Study was also chosen due to the fact that skeletal muscles cannot significantly recover from atrophy conditions following 48 h of mechanical loading^63^. In contrast to the overall telomere shortening of T cells in astronaut studies^26^, we found that the telomere length of skeletal muscle cells failed to return to baseline levels, despite the recovery of muscle mass.

The most common mechanism of telomere elongation is through the action of the enzyme telomerase^42^. A role for telomerase in spaceflight-induced telomere elongation has been suggested for peripheral T cells^26^. However, telomerase activity measurements in astronaut studies could not be measured midflight, as the activity of TERT in tissue samples was “lost in space”, likely hampered by the harsh conditions the samples experienced before returning to Earth^25,26,41^. In the mouse study presented here, TERT expression in skeletal muscles was low and coincided with low telomerase activity in our experimental groups. These findings are in agreement with a prior report that showed telomerase activity is lost in differentiated skeletal muscle cells^43^ and suggest that telomerase has minimal role in telomere elongation in this experimental paradigm. However, it cannot be ruled out that transient telomerase activity in small proportions of myogenic cells contributes to our observed telomere-lengthening phenotype. Our investigation into additional modes of telomere lengthening uncovered preliminary evidence of an alternative lengthening (ALT) mechanism engagement. ALT was originally discovered in immortalized cell lines and subsequently shown to occur in some human tumors^64,65^ While evidence is mounting that ALT can be regulated by environmental and genetic factors in rapidly proliferating tumor cells^44^, its modulation and engagement in quiescent non-cancerous cells has not been explored, particularly while maintaining tissue structure. In quiescent skeletal muscle myogenic cells, we found that there is a substantial downregulation of the chromatin remodeler *Atrx* and the histone chaperone *Daxx* in both the HLS + IR and the recovery muscles compared to controls. The biological underpinning of this downregulation in muscles remains unknown. Observed telomere lengthening, in conjunction with low telomerase activity and reduction of *Atrx* and *Daxx* expression, are suggestive of the engagement ALT-type mechanisms. In support of our findings, recent evidence has shown telomere lengthening in the blood cells of Mount Everest climbers during different points of peak ascent. Based on gene expression analyses, *TERT* was downregulated and *RAD50* was upregulated, while ALT and telomere maintenance via recombination pathways were enriched as the climbers progressed upwards^41^. These high-altitude studies, in which radiation exposure is higher than sea level, add weight to the validity of our findings of ALT-related pathway engagement over a relatively short time frame.

A conundrum of our findings relates to the fact that cells of undamaged adult skeletal muscles are quiescent, including the myogenic cells of our study. Since ALT utilizes some of the same players of homologous recombinational (HR) repair^66^, a DNA double-strand break repair mechanism that is most active during the S and G2 phases of the cell cycle^67^, our findings are unexpected. However, there is substantial evidence that HR can occur in the G1 phase of the cell cycle^68^ and in postmitotic neurons^69^. In addition, HR in general occurs at similar frequencies in ALT proficient and ALT-deficient human cells^70^, which adds an additional level of complexity to the understanding of ALT processes. Thus, it is therefore possible that telomerase-independent lengthening may be occurring in our experimental system, and that reduced expression of *Atrx* and *Daxx* supports the plausibility of a mechanism with features of ALT contributing to our observations. Until more details emerge on how molecular signatures associated with ALT are governed in nondividing, non-cancerous tissues, the specific mechanisms controlling telomere lengthening in a cell type-specific manner in our experimental design, or in the environment of space, remain elusive.

Overall, this study examined the relationship between skeletal muscle cells and telomere length dynamics following hindlimb suspension and irradiation. While these findings are not extendable to post-flight measurements of astronauts, the experimental design applied here can be of use for preassessments of space-determined factors that modulate telomere dynamics and skeletal muscle functionality. One limitation of our study is that we only use males. Recent studies have demonstrated no sex difference in adult telomere length in vertebrates^71^, thus including female mice in our study is unlikely to change the interpretation of the mouse results. Yet, studies in humans have revealed differences in telomere length between sexes, depending on the age examined and the methodology used^72–74^. Thus, subsequent studies should consider telomere differences between sexes, especially given the rise in the number of female astronauts participating in space missions, and other reported differences in additional metrics between the sexes in other spaceflight studies^75,76^. In any event, the data presented herein demonstrate that a ground-based model may be a useful starting point in elucidating the mechanisms governing telomere elongation dynamics in muscles of astronauts during spaceflight.

Another limitation of this study is the type and dose/dose-rate of radiation exposure and how it relates to astronauts’ occupational exposure. The space radiation environment is comprised of high energy protons and galactic cosmic radiation, including but not limited to electrons, protons, and heavier densely ionizing charged particles^77^. Additionally, radiation sources in space are not constant, and the absorbed dose varies between mice and humans varies, at least for proton radiation^78^. Our experimental outcome in ground-based rodent muscle utilized a single exposure to a single ionizing radiation source, as it was not feasible to reload rodent hindlimbs for possible successive radiation exposures. To ensure radiation exposures were sufficient during our experimental window, we utilized a radiation dose of 2 Gy, which at current standards, is higher than NASA occupational exposures^79^.

A major difference between mice and humans is telomere length, with many mouse strains having ∼8-fold longer telomeres than humans^80^, and they exhibit much faster rates of telomere shortening than humans^81^. This greater telomere reservoir could endow muscle cells in in-bred strains of laboratory mice with a protective effect in spaceflight, but it is equally as possible that telomere changes are more easily studied in rodent models because of their differences compared with humans. Skeletal muscle cell telomere length was not examined in astronaut studies^25,26,65^, and muscle biopsies of astronauts in space are rare, as the risk of complications to their health or possibly jeopardizing the missions is too great. Thus, future investigations using animal models in spaceflight would presumably mimic the muscle defects seen in astronauts and would be invaluable. Given the differences in telomere length between typically used research mouse strains and humans, future studies could be further strengthened by utilizing more humanized mice in regard to telomere length, such as the CAST/Ei J strain^82^. After being exposed to the same weightless and cosmic radiation conditions as humans, these mice may be useful to further investigate behavior and function of different cell types in relation to their telomere changes.

In conclusion, the data presented here lay a foundation for future studies to explore telomere length regulation in skeletal muscles during spaceflight conditions, to investigate whether ALT drivers exert cell type-specific influences on telomere length, and how long these changes last and how they contribute to health risks in astronauts after they return to Earth. Identifying the cellular and molecular muscle changes associated with telomere lengthening, as well as how ALT-related processes affect spaceflight-exposed cells, may yield important clues for future prophylactic therapy of astronauts’ muscles, both during space flights and upon return to Earth.

## Methods

### Mice

Male C57Bl/6 mice were purchased from Jackson Labs (stock# 000664) and used between 2 and 4 months of age. Experimental procedures for this project were approved and overseen by the University of Pennsylvania IACUC committee (protocol #806800). Following receipt, mice were acclimated to University of Pennsylvania rodent housing for 1 week prior to commencing the experiments. All mice in this study were fed Dietgel boost food (ClearH_2_O) and provided hydrogel barrier hydration (ClearH_2_O) ad libitum (as the sole nutrition and hydration sources) for the duration of the experiment.

### Full body irradiation

Cohorts of mice were irradiated with ionizing radiation from a ^137^Cs irradiator (Gammacell 40 exactor) at a dose-rate of 1.23 cGy/s. Room air was provided to the mice during the procedure via a vacuum pump. After 2 Gy exposure, mice were returned to conventional housing for 1-week, prior HLS.

### Hindlimb suspension

Equipment used for hindlimb suspension can be found in Supplementary Table 1. Briefly, mice were anesthetized with an isoflurane vaporizer (2.5% v/v isoflurane to oxygen), and mouse tails were wrapped with cloth surgical tape, beginning at the tail base. After wrapping ~half of the tail length, the tape was folded on itself to create a loop, and remaining tape was wrapped around the tail from the midpoint towards the tail base. Through this closed tape loop, plastic-coated wire was threaded and attached to a hanging apparatus. The hanging apparatus was placed in a rat cage with a wire mesh bottom. The hanging apparatus allows for movement of mice only through use of the forelimbs. Range of movement of HLS mice was approximately ½ of the rat cage surface area, but HLS mice were singly housed for the study. Mice were hung for a period of 3 weeks, with or without a 2-week recovery period where mice were placed in conventional housing.

### Tissue sample collection

At the end point of the study, mice were humanely euthanized by CO_2_ asphyxiation, followed by cervical dislocation. Organs of interest were collected by dissection and weighed. Tissue weights were normalized to endpoint mouse body weights. Gastrocnemius muscles were fixed in 4% paraformaldehyde in phosphate-buffered saline (PBS; Thermo) for 2 h on a rocking platform at 4 °C. Muscles were then incubated overnight in 30% sucrose in water at 4 °C, prior to embedding in OCT (NEG-50; Epredia) and cryofreezing. Muscle cryosections were cut at 10 μm and placed on superfrost plus slides (Fisher). Slides were stored at −20 °C prior to staining.

### Muscle fiber cross-sectional area measurements

Muscle cryosections were permeabilized with 0.5% triton X-100 for 10 min, washed with PBS, and blocked with 3% bovine serum albumin (BSA; GeminiBio) in PBS for 1 hour at room temperature. Slides were then incubated with Alexa Fluor 555-conjugated wheat germ agglutinin (WGA; 1 μg/mL; Thermo) for one hour in the dark at room temperature, to outline muscle fibers. Slides were washed in PBS and coverslips were mounted with prolong gold with DAPI (Thermo). Slides were imaged on a Nikon Ni widefield microscope equipped with a Plan Fluor 10x/0.30 objective and Nikon DS-Qi1Mc 14-bit camera, and random fields per mouse were used for fiber area enumeration in Fiji.

### Muscle stem cell number quantification

Muscle cryosections were permeabilized with 0.5% triton X-100 for 10 min, washed with PBS, and underwent heat-mediated antigen retrieval with EDTA buffer (1 mM EDTA, pH 8.0; 0.05% Tween 20). Slides were washed with PBS and blocked for 1 h at room temperature with 3% BSA/PBS/0.1% Triton X-100. Sections were stained with Pax7 rabbit polyclonal antibody (1/100; Thermo #PA1–117) overnight at 4 °C, washed with PBS, and stained with Alexa Fluor-647-conjugated Goat anti-rabbit IgG (1/300; Thermo) for 1 hour at room temperature. Slides were washed with PBS and coverslips were mounted with prolong gold with DAPI (Thermo). Whole cryosections were imaged using an Axio Scan z1 slide scanner (Zeiss) and processed with Zen software. Two cryosections per mouse were used for quantification of the number of pax7 positive cells per 10μm section with Fiji.

### Senescence-associated β-galactosidase activity assay

Cryosections from gastrocnemius muscles or mouse kidney were fixed with 1% paraformaldehyde/0.2% glutaraldehyde (Electron microscopy sciences). Slides were incubated with PBS, pH 6.0 for 30 minutes, followed by incubation with X-gal staining solution (4 mM potassium ferricyanide, 4 mM potassium ferrocyanide, 2 mM magnesium chloride, 0.02% Igepal CA-630, and 400 μg/mL X-gal; all from Sigma) for 48 h at 37 °C. Slides were washed with PBS, and fixed in 1% paraformaldehyde. Slides were washed with PBS and coverslips were mounted with Fluromount G (SouthernBiotech). Slides were imaged on a Nikon-Ni widefield microscope using a Nikon DS-F12 color camera.

### Telomere length assessments

Telomere length was measured using the CRYO MuQ-FISH protocol^35^. Briefly, muscle cryosections were permeabilized in 0.5% triton X-100 in PBS for 10 min, washed with PBS, and underwent heat-mediated antigen retrieval with TET buffer (10 mM Tris, pH 7.5, 1 mM EDTA, pH 8.0, 0.05% Tween-20) for 10 minutes. Slides were washed in PBS and incubated with RNase A (100 μg/mL) for 20 minutes at 37 °C. Slides were then incubated with Alexa Fluor 647-conjugated TelC telomere probe (1/600; PNA bio) in hybridization buffer [15% ethylene carbonate (Sigma), 20% dextran sulfate (Sigma), 600 mM sodium chloride (Sigma), 1X antigen retrieval citrate buffer (Vector labs)]. Hybridization occurred by a denaturation step of 67 °C for 10 min and a hybridization step of 42 °C for 90 min. A glass coverslip was used to prevent evaporation. Slides were washed in buffers with decreasing salt concentration (2X SSC; 0.1% Tween-20, 1X SSC; 0.1% Tween-20, 0.5X SSC; 0.1% Tween-20, 0.25X SSC; 0.1% Tween-20) twice each solution for 5 min each before a final rinse in PBS. Slides were then blocked with 3% BSA/PBS/0.1% triton X-100 for 1 hour. MuSCs were labeled by VCAM1 staining (1/100 of 1 mg/mL stock; Thermo # PA5–47029), endothelial cells were marked by CD31^biotin^ (1/100; Thermo #13–0311–82) antibody staining overnight at 4 °C. Alexa fluor 488-conjugated donkey anti-goat antibody (1/300; Thermo # A11055), or Cy3-streptavidin (1/100; Thermo) were used to label the cells of interest. After washing with PBS, glass coverslips were mounted with prolong gold with DAPI (Thermo). To image telomeres, MuSCs (VCAM1+ in the satellite cell position), myonuclei (based on position within the myofibers), or endothelial cells (CD31 + ) were imaged with a Nikon Plan Apo 100x/1.45 oil objective on a Nikon Ni widefield epifluorescence microscope, equipped with a DS-Q1MC monochrome camera. Z stacks of 1 μm thickness from each channel (telomere, DAPI, cell type) were taken to encompass the entirety of the telomere fields. Images were processed using the extended depth of focus algorithm in the accompanying Nikon elements microscope software. Images were exported as 16-bit monochrome tiffs and analyzed using the free analysis software, Telometer 3.0.6 (https://demarzolab.pathology.jhmi.edu/telometer/download.html). This software allows cells of interest to be analyzed for telomere signal intensities, and normalizes these intensities to that of DAPI, to account for differences in cell cycle position and has been previously validated for use in muscle.

### Quantitative real-time PCR

Total RNA was extracted from whole gastrocnemius muscles with Trizol. RNA was reverse transcribed into cDNA using the Protoscript II first strand cDNA synthesis kit. (NEB). Multiplexed quantitative real-time PCR was completed using either FAM-labeled *Tert* (ThermoFisher; gene assay id: Mm00436931_m1), *Atrx* (ThermoFisher; gene assay id: Mm00494196_m1), or *Daxx* (ThermoFisher; gene assay id: Mm00492089_m1), with VIC-labeled *Gapdh* (ThermoFisher 4352339e). Reactions were run on a Quantstudio 6 with accompanying software. Relative gene expression was determined by the ΔΔCT method, using *Gapdh* as a normalizing control.

### Immunohistochemistry

Gastrocnemius cryosections were permeabilized with 0.5% triton X-100 for 10 min, washed with PBS, and blocked with 3% bovine serum albumin (BSA; GeminiBio) in PBS for 1 h at room temperature. Primary antibody to TERT (1/100; RayBiotech # 144–64552–50) was incubated in blocking buffer overnight at 4 °C. Slides were washed the following day with PBS and stained with Alexa Fluor-conjugated goat anti-rabbit 555 secondary antibody (1/300; Thermo #A21428) at room temperature for 1 hour. Slides were washed with PBS and coverslips were mounted with prolong gold with DAPI (Thermo). Slides were imaged on a Nikon-Ni widefield microscope using a Nikon DS-F12 color camera.

### Telomerase activity measurement by TRAP assay

Relative telomerase activity was measured by Telomere Repeat Amplification Protocol (TRAP) assay as previously described^83^. In brief, muscle tissue was lysed in 1X CHAPS buffer for 30 minutes on ice. Lysates were then centrifuged at 16,000×*g* for 20 min to pellet cell debris and protein concentration was measured by Bradford assay. One hundred twenty-five nanograms of protein lysate was incubated with a telomerase substrate at 30 °C for 30 minutes to allow for telomerase to catalyze the addition of telomere repeats to the substrate. The reactions were then PCR amplified, resolved on a 4–20% TBE polyacrylamide gel at 200 V for 30 minutes and visualized by staining with SYBR Green nucleic acid gel stain. Relative telomerase activity was quantified using ImageJ software.

### Statistical analysis

Data were analyzed statistically using GraphPad (La Jolla, CA, USA) Prism 9.5.1 software. Results are presented as the mean± or SEM for column graphs, with individual data points representative of a single mouse dataset, or as median with 25th and 75th quartiles for violin plots. Datasets were analyzed with 1-way ANOVA to determine significance, with Tukey’s multiple comparison test of means used to obtain adjusted p values. Differences were considered statistically significant at *p* < 0.05. Sample sizes are indicated for each data set in the figure legends.

### Reporting summary

Further information on research design is available in the Nature Research Reporting Summary linked to this article.

### Supplementary information

Supplemental Material

Reporting Summary

## Data Availability

All data generated during and/or analyzed during this study are included in this published article and its supplementary information. Any detailed data supporting the findings of this study are available from the corresponding authors upon reasonable request.

## Code availability

Telometer telomere analysis software is available for free by the creators (https://demarzolab.pathology.jhmi.edu/telometer/).
