# Supplementary material for: Impacts of radiation exposure, hindlimb unloading, and recovery on murine skeletal muscle cell telomere length
Source: NPJ Microgravity. 2023 Sep 15;9:76. doi: 10.1038/s41526-023-00303-1 (PMC10504369; doi:10.1038/s41526-023-00303-1)
Supplement: Supplementary file 1 — Supplemental Material [file 41526_2023_303_MOESM1_ESM.pdf]

Impacts of radiation exposure, hindlimb unloading, and recovery on murine skeletal muscle cell telomere length

Elisia D. Tichy, Ji-Hyung Lee, Grant Li, Katrina N. Estep, F. Brad Johnson , and Foteini Mourkioti

Supplementary Figures and Tables

Supplementary Figures

Supplementary Figure 1

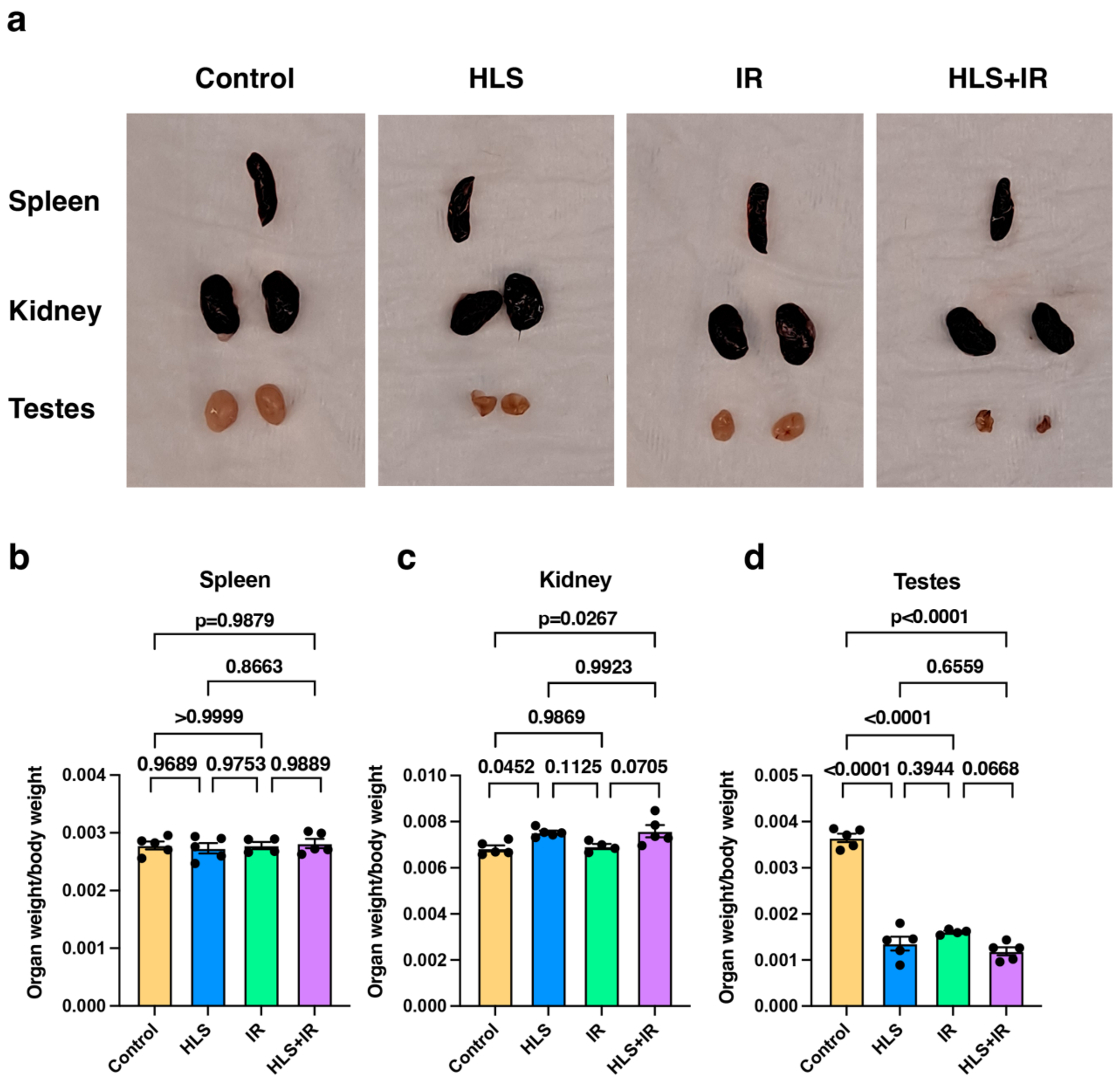

Supplementary Figure 1: Non-myogenic organ weights divided by mouse body weights. Individual organs were collected at the end of the 3-week study and weighed. Experimental groups included control (orange), hindlimb suspension (HLS; blue), irradiation treatment (IR; green), and a combination of HLS and IR (purple). Organ measurements included **a)** spleen, **b)** kidneys, and **c)** testes. Organ weights in grams were normalized to respective mouse body weights in grams. n=4-5 mice per condition. Displayed are mean ± SEM. Statistical analysis was determined by one-way ANOVA and Tukey's multiple comparison test of means. Adjusted p values are displayed.

Supplementary Figure 2

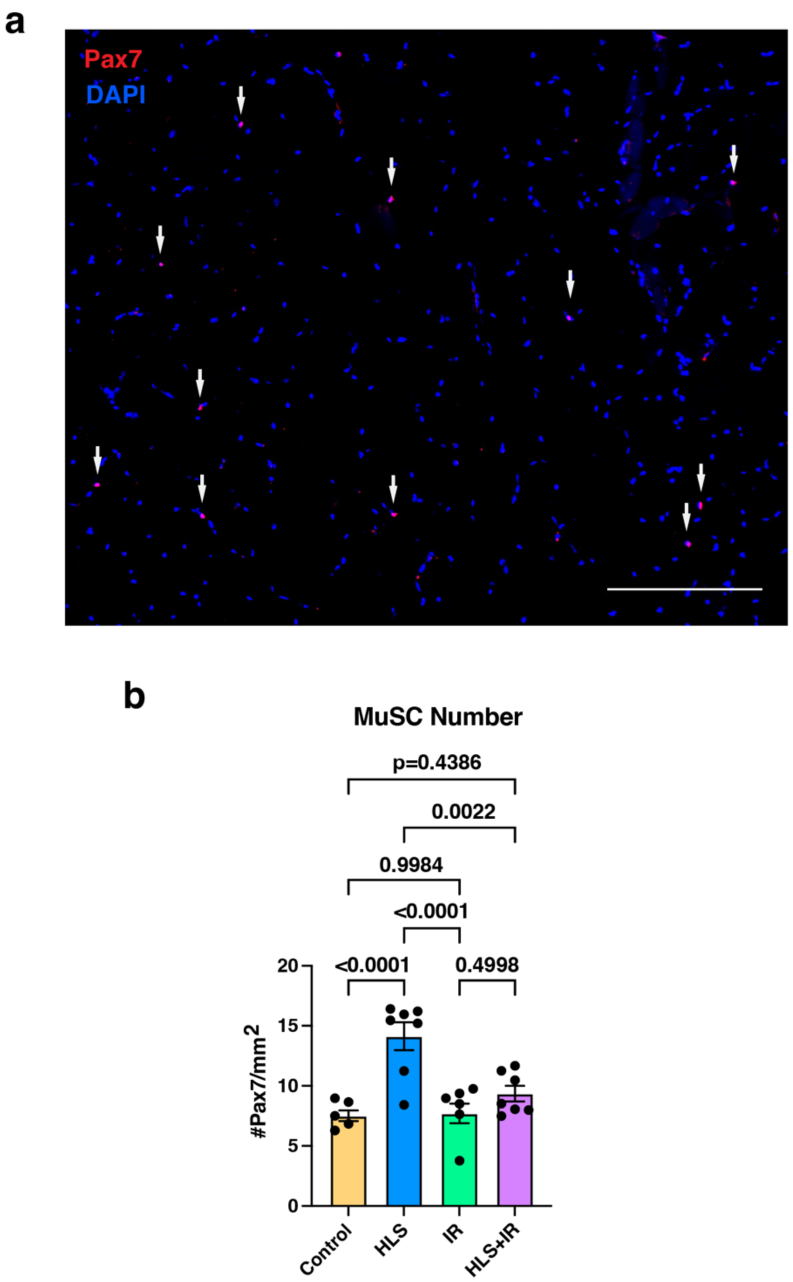

Supplementary Figure 2: MuSC number assessments in gastrocnemius muscles of experimental mice. a) Representative image of gastrocnemius cryosection stained with the MuSC marker, Pax7. Arrows represent positive cells. Scale bar: 200µm. **b)** Quantification of MuSC numbers per mm^2 in gastrocnemius cryosections. At least 5 mice were analyzed per condition. Displayed are mean ± SEM. Statistical analysis was determined by one-way ANOVA and Tukey's multiple comparison test of means. Adjusted p values are displayed.

Supplementary Figure 3

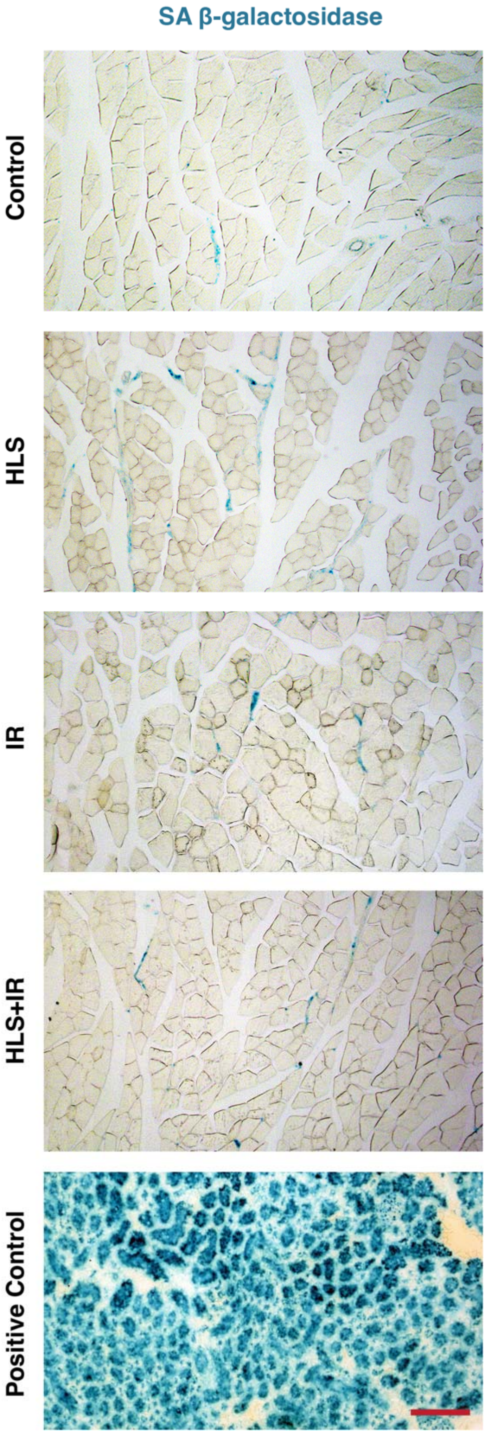

Supplementary Figure 3: Senescence activity measurements in experimental muscles. Cryosections from gastrocnemius muscles from control, HLS, IR, and HLS+IR mice were processed for senescence-associated beta-galactosidase activity (SA β-galactosidase). As a positive control, a kidney cryosection from aged mice was used. Scale bar: 100μm.

Supplementary Figure 4

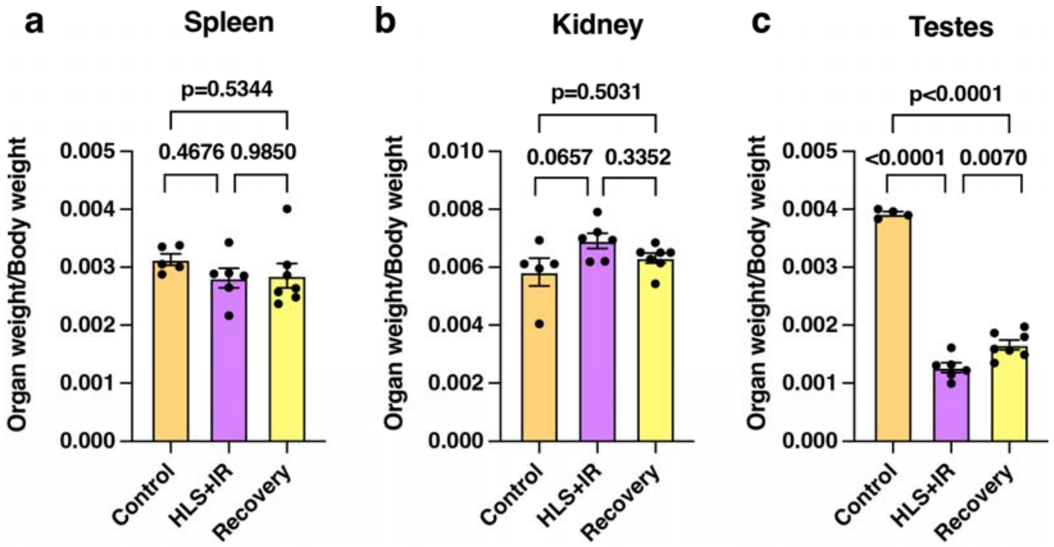

Supplementary Figure 4: Non-myogenic organ weights divided by mouse body weights. Individual organs were collected at the study endpoint and weighed. Experimental groups included control (orange), a combination of hindlimb suspension with Ionizing radiation treatment (HLS+IR; purple), and a HLS+IR with 2 week recovery group (yellow). Organ measurements included **a)** spleen, **b)** kidneys, and **c)** testes. Organ weights in grams were normalized to respective mouse body weights in grams. n=4-5 mice per condition. Displayed are mean ± SEM. Statistical analysis was determined by one-way ANOVA and Tukey's multiple comparison test of means. Adjusted p values are displayed.

Supplementary Figure 5

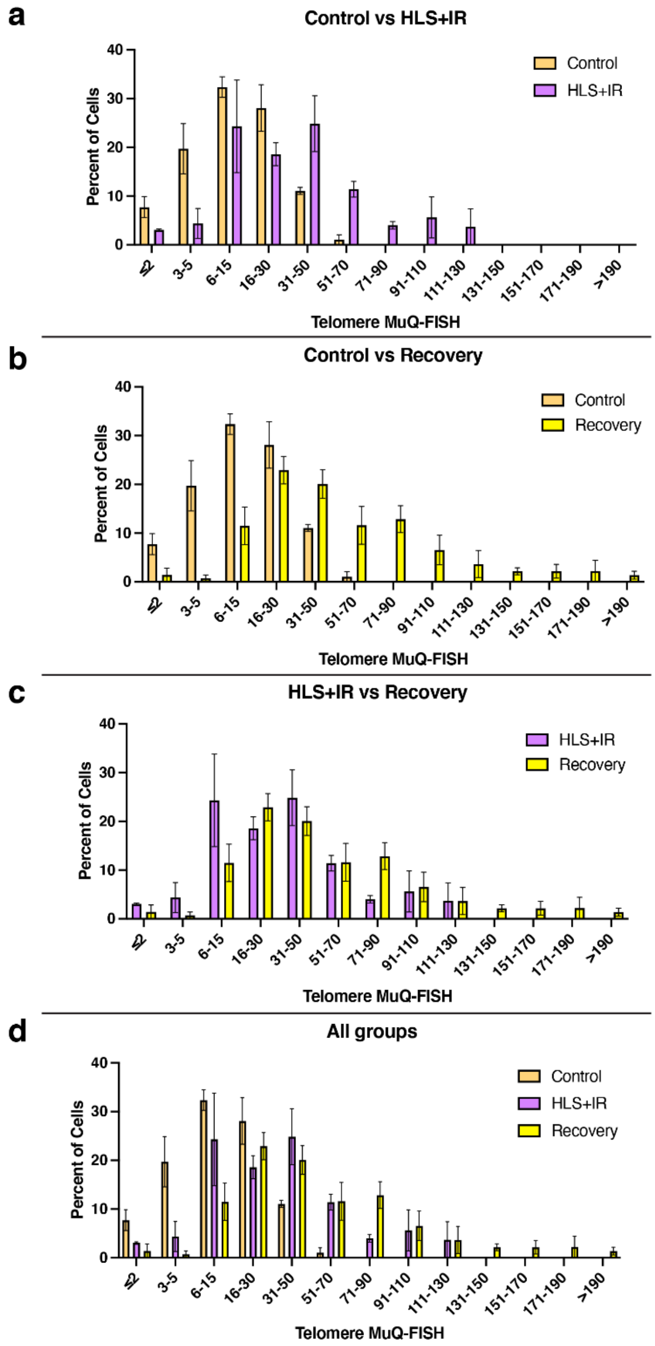

Supplementary Figure 5: Telomere length distributions reveal increased telomere lengths in experimental muscles. a) Comparison of telomere length by percent of MuSCs between control and HLS+IR groups. **b)** Comparison of telomere length by percent of MuSCs between control and recovery groups. **c)** Comparison of telomere length by percent of MuSCs between HLS+IR and recovery groups. **d)** Comparison of control, HLS+IR, and recovery MuSC groups in one graph for telomere length distributions on a per cell basis. At least 3 mice were analyzed per condition. Displayed are mean ± SEM.

Supplementary Figure 6

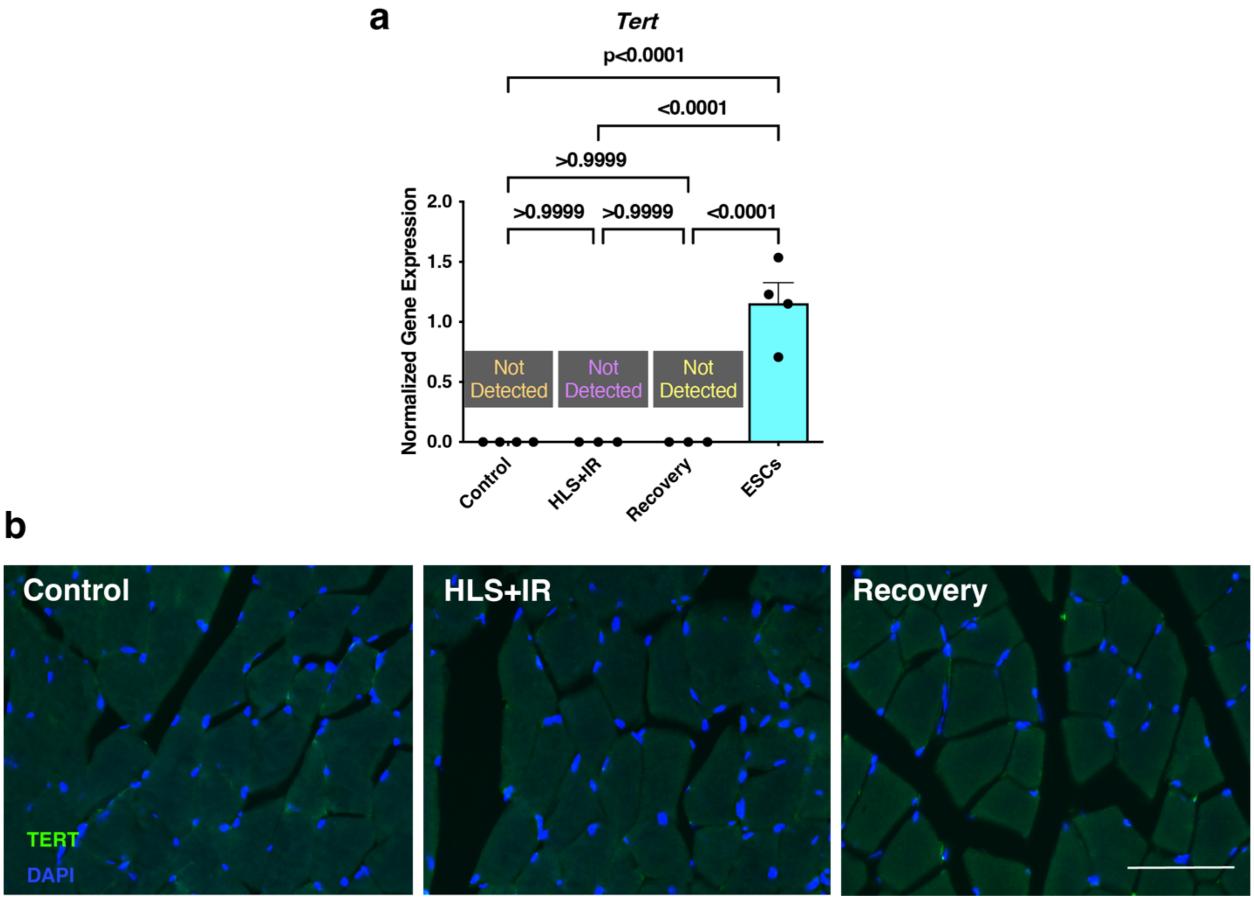

Supplementary Figure 6: Assessment of telomerase expression in control, HLS+IR, and recovery gastrocnemius muscles. a) Quantitative real-time PCR of control, HLS+IR, and recovery gastrocnemius muscles, examining expression of TERT. Gapdh was used as a housekeeping gene for normalization. Mouse embryonic stem cells served as a positive control. **b)** Immunohistochemistry of TERT in cryosections of control, HLS+IR, and recovery gastrocnemius muscles. Scale bar: 50μm. At least three animals were used per group. Statistical analysis was determined by one-way ANOVA and Tukey's multiple comparison test of means. Adjusted p values are displayed.

Supplementary Table

Supplementary Table 1: HLS equipment components

Item	Catalog Number	Supplier
Absorbent underpads with waterproof moisture barrier	56617-018	VWR
Bobbin, class 15	42136	Singer
Cap nut; #10-32	762406	Everbilt
Cloth tape; 1"	791-2PK	Nexcare
DietGel Boost	CW72-04-5022	ClearH2O (through Animal specialties and provisions distributor)
Fishing barrel swivel with nice snap	#8-100pcs	Shaddock Fishing
1 3/8" x 48" zinc plated punched steel flat bar 1/16" thick	584265	Everbilt
Hex nut; #10-32	800041	Everbilt
Hydrogel Barrier	CW70-01-5022	ClearH2O (through animal specialties and provisions distributor)
Lab tape, 3/4"	sc-224489	Santa Cruz Biotechnology
Mesh Caging Floor Insert	RAT-WIRE INSERT-DURA	Ares Distribution
Primary wire, 16 gauge	55668021	Southwire
Polycarbonate Rat Cage	RC88D-PC	Alternative Design Mfg & Supply Inc
Polycarbonate Rat Cage Filter Top	FT8XL-PC	Alternative Design Mfg & Supply Inc
2" thumb screw #10-32	B00HYK40D2	Hillman/Amazon
Washer; 1/4"	591378	Everbilt
Wing nut; #10-32	802361	Hillman
