# Supplementary material for: Impacts of radiation exposure, hindlimb unloading, and recovery on murine skeletal muscle cell telomere length
Source: NPJ Microgravity. 2023 Sep 15;9:76. doi: 10.1038/s41526-023-00303-1 (PMC10504369; doi:10.1038/s41526-023-00303-1)
Supplement: Supplementary file 2 — Reporting Summary [file 41526_2023_303_MOESM2_ESM.pdf]

nature portfolio

Corresponding author(s): Foteini Mourkioti

Last updated by author(s): Jun 26, 2023

Reporting Summary

Nature Portfolio wishes to improve the reproducibility of the work that we publish. This form provides structure for consistency and transparency in reporting. For further information on Nature Portfolio policies, see our Editorial Policies and the Editorial Policy Checklist.

Statistics

For all statistical analyses, confirm that the following items are present in the figure legend, table legend, main text, or Methods section.

n/a	Confirmed	
☐	☒	The exact sample size (*n*) for each experimental group/condition, given as a discrete number and unit of measurement
☐	☒	A statement on whether measurements were taken from distinct samples or whether the same sample was measured repeatedly
☐	☒	The statistical test(s) used AND whether they are one- or two-sided<br>*Only common tests should be described solely by name; describe more complex techniques in the Methods section.*
☐	☒	A description of all covariates tested
☐	☒	A description of any assumptions or corrections, such as tests of normality and adjustment for multiple comparisons
☐	☒	A full description of the statistical parameters including central tendency (e.g. means) or other basic estimates (e.g. regression coefficient) AND variation (e.g. standard deviation) or associated estimates of uncertainty (e.g. confidence intervals)
☐	☒	For null hypothesis testing, the test statistic (e.g. *F*, *t*, *r*) with confidence intervals, effect sizes, degrees of freedom and *P* value noted<br>*Give P values as exact values whenever suitable.*
☒	☐	For Bayesian analysis, information on the choice of priors and Markov chain Monte Carlo settings
☒	☐	For hierarchical and complex designs, identification of the appropriate level for tests and full reporting of outcomes
☒	☐	Estimates of effect sizes (e.g. Cohen's *d*, Pearson's *r*), indicating how they were calculated

Our web collection on statistics for biologists contains articles on many of the points above.

Software and code

Policy information about availability of computer code

Data collection	Images were collected using Nikon Elements software, QRT-PCR was collected by Quantstudio software
Data analysis	Telomere lengths were analyzed with telometer version 3.0.6 (https://demarzolab.pathology.jhmi.edu/telometer/download.html). Image quantifications not telomere-related were made with imageJ/Fiji.<br>Graphs were generated and statistical analysis was conducted with graphpad prism version 9.4.1.

For manuscripts utilizing custom algorithms or software that are central to the research but not yet described in published literature, software must be made available to editors and reviewers. We strongly encourage code deposition in a community repository (e.g. GitHub). See the Nature Portfolio guidelines for submitting code & software for further information.

Data

Policy information about availability of data

All manuscripts must include a data availability statement. This statement should provide the following information, where applicable:

- Accession codes, unique identifiers, or web links for publicly available datasets
- A description of any restrictions on data availability
- For clinical datasets or third party data, please ensure that the statement adheres to our policy

No public datasets were utilized and no data required deposition into public databases.

Human research participants

Policy information about studies involving human research participants and Sex and Gender in Research.

Reporting on sex and gender	This study includes no human subject data.
Population characteristics	This study includes no human subject data.
Recruitment	This study includes no human subject data.
Ethics oversight	This study includes no human subject data.

Note that full information on the approval of the study protocol must also be provided in the manuscript.

Field-specific reporting

Please select the one below that is the best fit for your research. If you are not sure, read the appropriate sections before making your selection.

☒ Life sciences ☐ Behavioural & social sciences ☐ Ecological, evolutionary & environmental sciences

For a reference copy of the document with all sections, see nature.com/documents/nr-reporting-summary-flat.pdf

Life sciences study design

All studies must disclose on these points even when the disclosure is negative.

Sample size	No sample size calculation was performed. Sample sizes were chosen based on the type of experiment, availability of animals, and standard practice when using individual animals as biological replicates (general range n=3-5).
Data exclusions	No data were excluded from the analysis.
Replication	We analyzed biological (not technical) replicates to ensure reproducibility. Experiments were reproducible between investigators involved.
Randomization	Mice were randomly assigned to different conditions, but no attempt was made to control for any other covariates.
Blinding	Investigators were blinded during all image analysis. Clear cutoffs and automated analyses were used to minimize the effects of investigator bias.

Reporting for specific materials, systems and methods

We require information from authors about some types of materials, experimental systems and methods used in many studies. Here, indicate whether each material, system or method listed is relevant to your study. If you are not sure if a list item applies to your research, read the appropriate section before selecting a response.

Materials & experimental systems

n/a	Involved in the study	
☐	☒	Antibodies
☒	☐	Eukaryotic cell lines
☒	☐	Palaeontology and archaeology
☐	☒	Animals and other organisms
☒	☐	Clinical data
☒	☐	Dual use research of concern

Methods

n/a	Involved in the study	
☒	☐	ChIP-seq
☒	☐	Flow cytometry
☒	☐	MRI-based neuroimaging

Antibodies

Antibodies used

anti-VCAM1, Thermo PA5-47029 Immunohistochemistry Goat polyclonal 1/100 of 1ug/uL
anti-Pax7 Thermo PA1-117 Immunohistochemistry Rabbit polyclonal 1/100
anti-CD31-biotin Thermo 13-0311-82 Immunohistochemistry Rat polyclonal 1/100
anti-Tert Raybiotech 144-64552-50 Immunohistochemistry Rabbit polyclonal 1/100
Donkey anti-goat IgG Alexa Fluor 488 A11055 1/300

	Goat anti-rabbit IgG Alexa Fluor 555 A21428 1/300<br>Cy3-streptavidin Thermo 434315 1/100
Validation	anti-VCAM1 was validated by the manufacturer and specificity was demonstrated in Rader Aging Cell 2018.<br>anti-Pax7 was validated by the manufacturer in C2C12 mouse myoblasts and characterized in Rozo Nature Medicine 2016.<br>anti-CD31-biotin was validated by the manufacturer and demonstrated specificity in Gromova Bio-Protocol 2015.<br>anti-Tert was validated by the manufacturer and is recommended for IHC.<br>Donkey anti-goat IgG Alexa Fluor 488 was validated by the manufacturer.<br>Goat anti-rabbit IgG Alexa Fluor 555 was validated by the manufacturer.<br>Cy3-streptavidin was validated by the manufacturer.

Animals and other research organisms

Policy information about studies involving animals; ARRIVE guidelines recommended for reporting animal research, and Sex and Gender in Research

Laboratory animals	Wildtype C57Bl/6 mice from the jackson laboratory (stock #000664).
Wild animals	No wild animals were included in this study.
Reporting on sex	Only males were included in the study design.
Field-collected samples	No field-collected samples were included in this study.
Ethics oversight	Experiments conducted on mice were approved by the University of Pennsylvania IACUC; protocol #806800.

Note that full information on the approval of the study protocol must also be provided in the manuscript.
